# A new rapid kindling variant for induction of cortical epileptogenesis in freely moving rats

**DOI:** 10.3389/fncel.2014.00200

**Published:** 2014-07-23

**Authors:** Juan Carlos Morales, Carla Álvarez-Ferradas, Manuel Roncagliolo, Marco Fuenzalida, Mario Wellmann, Francisco Javier Nualart, Christian Bonansco

**Affiliations:** ^1^Centro de Neurobiología y Plasticidad Cerebral, Facultad de Ciencias, Instituto de Fisiología, Universidad de ValparaísoValparaíso, Chile; ^2^Laboratorio de Neurobiología y Células Madre, Departamento de Biología Celular, Universidad de ConcepciónConcepción, Chile

**Keywords:** chronic-epilepsy, kindling, cortical-EEG, hippocampal-formation, stereotaxic-surgery

## Abstract

Kindling, one of the most used models of experimental epilepsy is based on daily electrical stimulation in several brain structures. Unlike the classic or slow kindling protocols (SK), the rapid kindling types (RK) described until now require continuous stimulation at suprathreshold intensities applied directly to the same brain structure used for subsequent electrophysiological and immunohistochemical studies, usually the hippocampus. However, the cellular changes observed in these rapid protocols, such as astrogliosis and neuronal loss, could be due to experimental manipulation more than to epileptogenesis-related alterations. Here, we developed a new RK protocol in order to generate an improved model of temporal lobe epilepsy (TLE) which allows gradual progression of the epilepsy as well as obtaining an epileptic hippocampus, thus avoiding direct surgical manipulation and electric stimulation over this structure. This new protocol consists of basolateral amygdala (BLA) stimulation with 10 trains of biphasic pulses (10 s; 50 Hz) per day with 20 min-intervals, during 3 consecutive days, using a subconvulsive and subthreshold intensity, which guarantees tissue integrity. The progression of epileptic activity was evaluated in freely moving rats through electroencephalographic (EEG) recordings from cortex and amygdala, accompanied with synchronized video recordings. Moreover, we assessed the effectiveness of RK protocol and the establishment of epilepsy by evaluating cellular alterations of hippocampal slices from kindled rats. RK protocol induced convulsive states similar to SK protocols but in 3 days, with persistently lowered threshold to seizure induction and epileptogenic-dependent cellular changes in amygdala projection areas. We concluded that this novel RK protocol introduces a new variant of the chronic epileptogenesis models in freely moving rats, which is faster, highly reproducible and causes minimum cell damage with respect to that observed in other experimental models of epilepsy.

## Introduction

Epilepsy encloses a set of neurological disorders of diverse etiology, characterized by the development of gradual and progressive spontaneous seizures, which increase in recurrence and severity with time. Abnormal synchronous hyperexcitability of a neuronal population underlies the epileptic seizure and may compromise cortical and subcortical structures, which are responsible for the typical epileptic symptoms (Gastaut, 1973; McIntyre et al., 2002; Morimoto et al., 2004). To study epilepsy, several *in vitro* and *in vivo* models have been developed. Most of the *in vivo* experimental epilepsy models have been based on pharmacological manipulations (i.e., chemical-kindling) (Dhir, 2012) or electrical stimulation (Morimoto et al., 2004; McNamara et al., 2006).

Kindling remains one of the few chronic models available to study epilepsy development (i.e., epileptogenesis) (Goddard, 1967, 1983; Goddard et al., 1969; Racine, 1972b; Lothman et al., 1985). Moreover, kindling allows precise control of epilepsy progression and epileptogenic focus (Goddard et al., 1969), which represent an advantage regarding other epileptogenesis models, including chemical-kindling, trauma-induced epileptogenesis or genetic models (Verma-Ahuja et al., 1995; D’Alimonte et al., 2009; Rakhade and Jensen, 2009). The conventional kindling protocol consists of repetitive subconvulsive electrical stimulation that elicits gradual and progressive enhancement of electroencephalographic (EEG) activity and behavioral responses, culminating in generalized seizures (Goddard et al., 1969; Racine, 1972b). EEG activity induced in the stimulated structure—namely afterdischarge (AD)—has been correlated with an increase in synchronous activity and hyperexcitability of a large group of neurons (Taylor and Dudek, 1982). The protocol progression is assessed through a behavioral scale known as “Racine Stages” (Racine, 1972b), which can be directly correlated with the AD duration and EEG frequency bands (Musto et al., 2009; Tsuchiya and Kogure, 2011).

Classical kindling protocols require a long period of stimulation (up to 30 days) applied directly to the amygdaloid complex (Behr et al., 2000; Von Bohlen und Halbach et al., 2004). Several variants of the classic protocol have been developed (Gersch and Goddard, 1970; Racine, 1972b; Lothman et al., 1985; Fournier et al., 2010) by changing the stimulation site and/or by adjusting stimulation parameters (i.e., intensity, pulse duration, phase, frequency, stimuli train duration and inter-stimulus interval). Regarding these parameters, kindling protocols can be classified in slow (SK; Goddard, 1967; Racine, 1972b; Dragunow et al., 1985) and rapid (RK; Gilbert and Cain, 1981; Lothman et al., 1985; Rempe et al., 1997; Musto et al., 2009), depending on the number of stimulation days required to obtain fully kindled rats, which display consecutive generalized seizures (i.e., kindled state).

SK protocols require at least 16 days of daily subthreshold stimulation (i.e., below the intensity needed to generate an AD) to reach kindled state, while RK protocols require a maximum of 4 days with variable number of stimuli per day at suprathreshold (i.e., above the intensity needed to generate an AD) stimulation intensities (Gilbert and Cain, 1981; Lothman et al., 1985; Elmér et al., 1998). RK protocols developed until now present several limitations: (1) suprathreshold intensity stimulation; (2) lack of a gradual progression of ADs duration, recurrence and/or severity of seizures; and (3) direct stimulation on the structure of interest (i.e., cortical areas and hippocampal formation (Lothman et al., 1985). These limitations restrict the use of available RK protocols as models for ictogenesis more than epileptogenesis.

In this study, we developed a new variant of RK protocol to solve previous limitations by modifying stimulation parameters, thus allowing a gradual and reproducible progression of convulsive behavior (i.e., Racine stages) and epileptic EEG activity within 3 days, alterations that persist for at least one month. We achieved that by employing subthreshold stimulus intensity applied to the basolateral amygdala (BLA). Moreover, the direct stimulation of the amygdala generates an epileptic hippocampus, which exhibits some of the epilepsy-related pathophysiological key features (De Lanerolle et al., 2012) without surgical procedures and direct stimulation to the hippocampus. Thus, this protocol provides an accurate experimental tool to study the cellular mechanisms of epilepsy development and establishment.

## Materials and methods

### Animals

Male rats of the Sprague Dawley strain (250–320 gr.) were used. The procedures of animal care, surgery and recording were in accordance with the guidelines laid down by the Institutional Animal Care and Ethics Committee at the Faculty of Sciences, Universidad de Valparaiso and NIH, USA. The animals were fed *ad libitum* in a 12:12 h light/dark cycle at 21 ± 2°C with a relative humidity of 55 ± 5%. Once the animals were selected, they were housed individually in transparent plastic cages during all handling, transportation and EEG recording procedures. Efforts were made to minimize the number of animals used and their suffering.

### Stereotactic surgery

Rats were sedated with a mixture of Ketamine 10% (57.0 mg/Kg, Troy Laboratories, Australia) and xylazine 2% (9.0 mg/Kg, Laboratorio Centrovet Ltda., Chile) administered through an intraperitoneal injection and subsequently, placed in a stereotactic apparatus (David Kopf, model 900). The surgical procedures for electrode implantation were carried out as described (Greenwood et al., 1991; Corcoran et al., 2011), with minor modifications. The skull was leveled and a teflon insulated bipolar stimulating and recording electrode of twisted stainless steel (0.2 mm of outer diameter, Plastics One Inc.) implanted in the right BLA to deliver kindling stimulation and record the ADs after the stimulus ended. The coordinates for BLA were calculated respect to Bregma: AP −2.12 mm, ML 5.0 mm and DV −8.0 mm. The placement of the intracranial electrode and their correct position was checked by standard histological verification in five rats per group, whose brains were fixated via intra cardiac perfusion with 4% paraformaldehyde (PFA) for later *Nissl* staining. Two pairs of stainless steel screws (≈1.45 mm in diameter) were used as epidural electrodes for EEG cortical recording and were bilaterally implanted on primary motor cortex M1 (Coordinates from Bregma: AP −2.12 mm, ML 2.0 mm and DV −1.5 mm) and on secondary visual cortex V2MM (respect to Bregma: AP −6.12 mm, ML 2.5 mm, DV −1.5). The V2MM ipsi and contralateral electrodes were used as differential signals for M1 ipsi and contralateral electrodes, respectively. All coordinates were obtained from the Paxinos and Watson (1998). All electrodes were fixed with dental acrylic and its terminals inserted in a plastic socket of six cavities, connected to headstage through a multichannel commutator with a balance rotor (Plastics One Inc., USA). All EEG records and behavioral manifestation monitoring were done in freely moving rats. All surgical materials were disinfected with Chlorhexidine Gluconate 2% topical solution (Difempharma). After surgery, the rats were allowed a 1-week recovery period before the start of the kindling procedure.

### Kindling procedures

**Figure 1:**
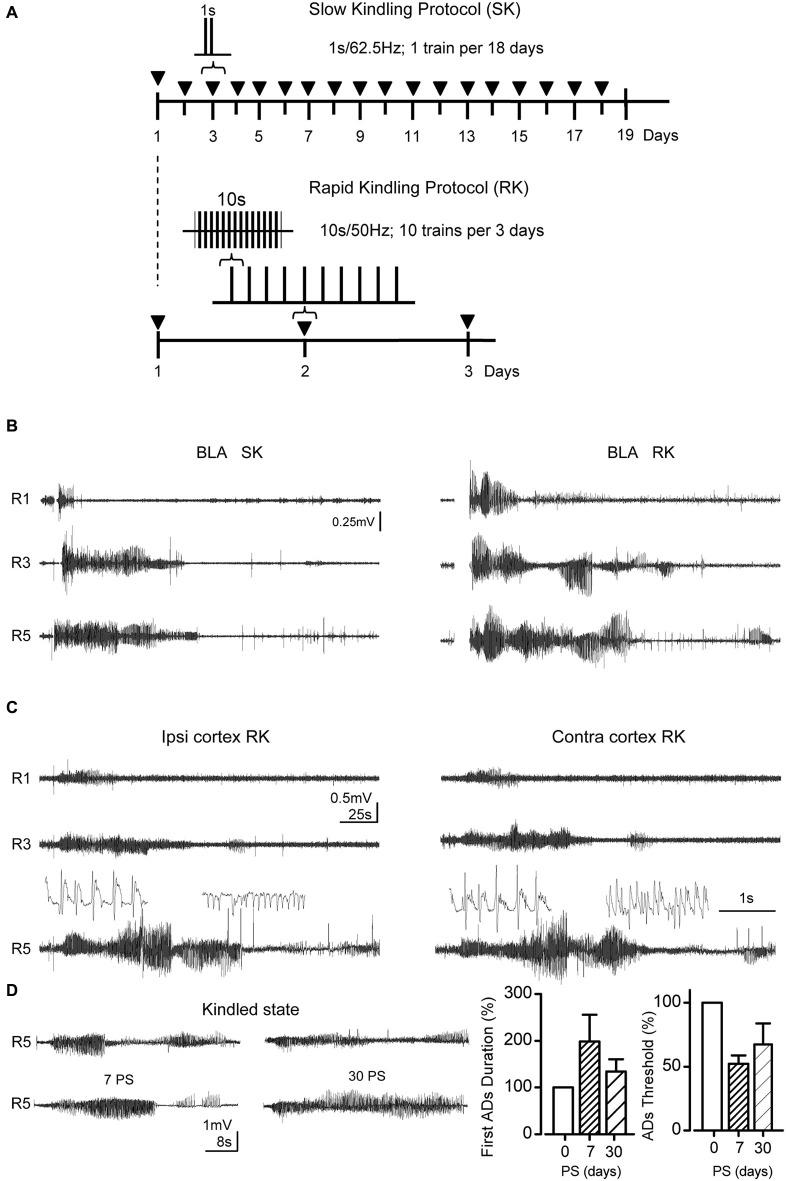
**RK protocol reproduces the after discharges (ADs) patterns evoked by SK protocol. (A)** Temporal course diagram for both protocols, where ▼ represents 1 stimulation session. In SK protocol 1 s train of monophasic pulses at 62.5 Hz during 1 s is applied in each session, whereas in RK 10 biphasic pulse trains at 50 Hz, 10 s each, were applied with 20 min inter-stimuli interval. For both trains, square pulses (1 ms of duration) were used. **(B)** Representative ADs from the basolateral amygdala (BLA), during Racine stages 1, 3 and 5. **(C)** ADs recorded simultaneously in motor cortex and BLA evoked by RK protocol in B. The insets show spike-waves and heterogeneous spikes from R5 ADs (below) in an expanded scale. **(D)** Representative ADs recordings corresponding to R5 at 0, 7 and 30 post-stimulation days (0, 7 and 30 PS) after RK protocol completion. Left, bar charts represent the percent change of the R5 first-AD duration with respect to 0 PS ADs (during protocol) at the indicated days. Right, percentage change of ADs thresholds at 0, 7 and 30 PS (*n* = 6 at 0, *n* = 3 at 7 and 30 PS, non-overlapping groups).

We compared the temporal course and induction properties of our RK kindling protocol, applied for 3 days, with respect to SK protocol, applied for 18 days. Additionally, the RK protocol was retested a week and a month after kindled state had been reached. These two protocols differ in the following aspects: frequency of pulses, phase of pulses, duration of train, number of trains and the intensity of the current applied in each pulse (Figure 1A, Table 1). Then, we studied four groups of animals, referred to as: the RK, SK, sham (operated but not stimulated), and control group (without surgical interventions).

**Table 1:** **Protocol parameters and outcome comparison between RK and SK**.

	**Protocol parameters**	
	RK	SK
Number of Days	3	17
Trains per Day	10	1
Inter-Stimulus Interval	20 m	24 h
Stimulus duration (train duration)	10 s	1 s
Train frequency	50 Hz (biphasic)	62.5 Hz (monophasic)
Train pulses width	1.0 ms (square)	1.0 ms (square)
	**Protocol outcome**	
	RK	SK
Total generalized seizures	3.6 ± 0.8 R4	4.8 ± 0.8 R4
	6.4 ± 0.8 R5	7.8 ± 0.8 R5
First AD duration	46.5 ± 5.2 s (R4)	46.4 ± 4.9 s (R4)
	62.3 ± 3.8 s (R5)*	48.7 ± 3.6 s (R5)
Trains needed to reach R4 and R5	9.8 ± 2.8 R4	4.3 ± 0.3 R4
	12.3 ± 1.4 R5*	6.4 ± 0.4 R5
Initial AD thresholds	73.5 ± 13.7 μA*	260 ± 24.5 μA

t-test *p < 0.05.

#### After discharge threshold

We identify the ADs as high amplitude EEG waves (i.e., ≥750 μV peak to peak) that appear in the form of spikes trains and spike-waves most of them between 1–8 Hz, and lasting for at least 5 s (Lothman et al., 1985; Musto et al., 2009; Tsuchiya and Kogure, 2011). In both RK and SK groups, the AD threshold, described as the minimum current intensity needed to evoke an AD in any recording channel; was determined for each rat. For the RK protocol, we increased current intensity by a factor of 1.25 every 10 min, starting at 10 μA until the appearance of the first AD. A similar procedure was used for SK protocol, but with an initial intensity of 100 μA. Differences in initial stimulus intensities were justified by train durations (RK: 10 s; SK: 1 s). Once AD threshold was identified, the stimulus intensity was set 20% below, this being completely innocuous and subthreshold at the beginning of the stimulation protocol. The animals that displayed AD thresholds over 200 μA and 400 μA in RK and SK protocols respectively were excluded due to probable defects in the implanted electrodes or surgical procedures. The same procedure was used for retesting the permanence of the alterations.

#### Kindling stimulation protocols

**(A)** For RK protocol, rats were stimulated during 3 days. RK stimulation trains consisted of biphasic square current pulses (1 ms) of 10 s at 50 Hz, delivered 10 times per day every 20 min (Figure 1A, Table 1). The alternating polarities of the pulses were used to reduce the capacitive attenuation of current during the train. Every kindling session were carried out between 10:00 and 16:00 h, starting at the same hour for each rat (i.e., 4 h top per session). Rats kept their sleep-wake cycle. Control recordings (20 min) were carried out every day previous to the kindling protocol to check the electrodes. Then, the protocol began and the first stimulus was delivered at 20 min, providing a 40 min habituation period in total every day. A retesting procedure was applied post stimulation at days 7 and 30. Firstly, we re-evaluated the AD threshold; and secondly, we verified the persistence of the kindled state evoked by three trains using the initial subthreshold stimulation applied to each rat.

**(B)** For SK protocol, rats were stimulated for at least 18 days. SK stimulation consisted of one train per day, which was composed of monophasic square current pulses (1 ms). The train had a 1 s-duration at 62.5 Hz (Figure 1A). In this case, due to the short train duration, the capacitive attenuation was not present.

### Electroencephalographic recordings and behavioral studies

The EEG activity from BLA, was recorded through an analog differential amplifier (AM-Systems, model 1700) 1000 × gain, filtered at 1–500 Hz, and digitalized at 1 kHz frequency sampling in a PowerLab bioamplifier (4/25T model), low pass filtered at 20 Hz, with a range of 500 mV single signal and divided by 1000. Cortical electrodes (M1 and V2MM) were directly connected to the same PowerLab via differential amplifier input with a range of 2 mV, and 1 kHz sampling and filtered between 1–50 Hz. All EEG recordings were done by using LabChart v7 software, and analyzed on-line by means of fast fourier transform (FFT) spectral analysis extension (size: 4 K; Data window: Blackman; Window overlay 93.75%; zero frequency was removed; display mode: amplitude).

The convulsive progression was evaluated according to Racine stages of epileptic seizures via direct inspection, and corroborated by posterior analysis of video recordings. The hallmarks of the Racine stages used were: oro-alimentary gesticulation (R1), head nodding (R2), unilateral forelimb clonus (R3), bilateral forelimb clonus and rearing (R4), and loss of postural control and tonic-clonic contractions in all limbs (R5). Every Racine stage includes signs of the previous one. The protocol concluded when the rats underwent three or more R5 seizures. In these conditions, the rat was considered in kindled state (Racine, 1972b). Normal behavior is scored as R0. ADs parameters were measured and recorded with the corresponding Racine stage for posterior data analysis.

#### Slice preparation

In order to evaluate the propagation of epileptiform activity to subcortical structures from BLA projections, we studied the electrical properties of CA1 pyramidal neurons in transversal hippocampal slices obtained from control and RK group. In this subset of experiments, the brains were removed by craniotomy and transversal slices were obtained to preserve the trisynaptic hippocampal circuit. The cutting was performed in cold artificial cerebrospinal fluid (ACSF; <2°C) using a Vibroslice microtome (i.e: 350 μm; NVSLM1, WPI, Inc.). After that, each slice was immediately taken to a chamber with ACSF at room temperature (20°C) for an incubation of 1 h. ACSF is bubbled with carbogen gas (95% O_2_, 5% CO_2_). The composition of the ACSF was (mM): 124.0 NaCl, 2.7 KCl, 1.25 KH_2_PO_4_, 1.3 Mg_2_SO_4_, 26.0 NaHCO_3_, 2.5 CaCl_2_ and 10.0 glucose, at pH = 7.4 (Bonansco and Buño, 2003). The recording was carried out in a recording chamber with gasified ACSF perfusion system (2 mL/min) attached to a Nikon microscope (FN 100 IR).

#### Electrophysiological recordings

Intracellular recordings of pyramidal neurons were made in the CA1 region of the hippocampus. Cell recordings were performed through whole-cell configuration with fire-polished pipettes (3–5 MΩ) filled with intracellular solution (see below), connected to an EPC-7 patch-clamp amplifier (Heka Instruments), filtered at 3.0 kHz, sampled at 4.0 kHz using an A/D converter (ITC-16; Intrutech) and stored with Pulse FIT software (Heka Instruments). Single electrode current-clamp recordings were obtained from pyramidal neurons of CA1, at a holding potential of −70 mV, unless otherwise specified. The intracellular solution contained (mM): 97.5 K-Gluconate, 32.5 KCl, 10.0 HEPES, 1.0 MgCl_2_ 6H_2_O, 5.0 EGTA, and 4.0 Na_2_-ATP, at pH = 7.2. Experiments started after a 5–10 min stabilization period following the establishment of whole-cell configuration.

#### Immunohistochemistry and confocal microscopy

Samples of transverse slices were obtained from control and sham, and those from kindled groups were obtained between 2 and 5 days after protocol finished. Samples were fixed in 4% PFA for 12 h. For the immunohistochemical analysis, the slices were incubated with anti-GFAP (1:200, DAKO) diluted in Tris-phosphate buffer and bovine serum albumin 1% p/v, overnight (Cortés-Campos et al., 2011). The samples were then incubated with Cy2-conjugated rabbit anti-IgG (Jackson Immuno Research, Pennsylvania, USA) for 2 h at 22°C. Hoechst was used for nuclear staining. The images (512 × 512 × 8 bits or 1024 × 1024 × 8 bits) were obtained using a confocal spectral microscope Zeiss 780. Quantification of hippocampal GFAP and Hoecht staining was assessed by using the Imaris software (Bitplane AG, Swizerland) and Image J (NIH, USA). Representative images were obtained using a known area. Results are expressed as the inmunostaining relative area (i.e., the area of individual units of staining) and the average inmunostaining intensity (expressed in Arbitrary Units, AU). A subset of samples was used to assess astroglial morphology in the hippocampus, using specific dye marker of protoplasmatic astrocytes Sulforhodamine 101 (SR101; Nimmerjahn et al., 2004). Acute slices were incubated with red fluorescent dye SR101 (0.5–1 μM) for 20 min in low Ca^2+^—high Mg^2+^ ACSF at 32–34°C (4.0/1.0 mM, respectively). Samples were visualized using both confocal and fluorescence microscopy, and the images were quantified in the same way as in immunohistochemical experiments.

### Parameters and data analysis

For both kindling protocols the duration of the first AD, the number of ADs per train and seizure total duration was measured. Because it has been suggested that the recovery time of neuronal activity between seizures may be determinant for the occurrence of both ADs and behavioral manifestation during consecutive stimulations (Goddard et al., 1969), inter train interval (ITI) was considered as a limiting parameter to the kindling progression. Consequently, to measure the evolution of Racine stages during the RK protocol we considered only those states equal to or greater than those previously observed. This was not necessary for SK protocols, since the ITI was sufficiently prolonged (24 h in SK with respect to 20 min in RK) to allow a sustained increase of the behavioral Racine stages.

Firstly, AD duration was considered as a central parameter because seizures were only observed during this period. Those recorded in BLA were measured from the end of the stimulus due to occlusion of the stimulation artifact. In M1 and VMM2 recordings, the first AD duration was measured directly from the start of the spike burst until reaching the baseline. Total seizure duration (TSD) was measured from the beginning of the first AD until the end of the last secondary AD.

Electrophysiological recordings were analyzed off-line through ClampFit 10.0. The data analysis was performed with Origin 6.0 software. Data was analyzed to determine if it fitted normal distribution (Shapiro-Wilk test). In that respect, a parametric (Student’s two-tailed *t*-test) or a non-parametric test (Mann-Whitney test) was performed as indicated. Differences were considered statistically significant at *p* < 0.05.

## Results

### Changes in EEG recordings from BLA and M1 induced by SK and RK protocols

In order to compare the progression of epileptiform activity induced by our RK variant with regards to traditional SK protocols, we assessed the electrical activity evoked in the BLA. Remarkably, the main modifications introduced in RK protocols were: ITI reduction (20 min in RK with respect to 24 h in SK); train duration increment (10 s in RK vs. 1 s in SK), and biphasic vs. monophasic stimuli (RK and SK, respectively; Figure 1A). As shown in Figure 1B, in both protocols, the stimulation of BLA, which is initially subthreshold and innocuous, induced the emergence of bursts of spike-waves patterns with high amplitude and low frequencies as the protocol progresses, which correspond to ADs, in both BLA and M1 cortex (Figures 1B,C, inset). In both groups, the first AD was always accompanied with a clear behavioral manifestation that could be classified by the Racine scale. In the first stimulations, ADs were brief and variable in both amplitude and frequency (Figures 1B,C, R1). The AD amplitude and duration increased progressively with repeated stimulations, together with the seizure severity (Figures 1B,C, R3). In more advanced stages (i.e., R4 and R5), both stimulation protocols were able to evoke a variable number of ADs, namely secondary ADs, whose amplitude and duration were lower than the initial one (Figures 1B,C, R5). Most severe seizures showed bilateral forelimb clonus and loss of postural control along with tonic-clonic contractions in all limbs.

In RK protocols, ADs waveform recorded in R5 were composed of bursts of heterogeneous spike-waves (Figure 1C, insets), predominantly in range of 1–4 Hz (δ-waves) and 5–8 Hz (θ-waves), whose mean spectral amplitudes were 52.4 ± 6.9 μV, and 22.2 ± 2.1 μV, respectively. These ranges were similar to those obtained in SK protocols. In these conditions, kindled state was considered when at least three consecutive R5 occurred. In RK protocol, the R5 can be elicited by a single stimulus using the initial intensity 7 and 30 days post stimulation (i.e., 7 PS and 30 PS; Figure 1D); moreover, ADs threshold decreased after the protocol completion, remaining lowered at 7 and 30 days PS (Figure 1D), being in accordance with the persistence of kindled state induced by SK protocol described by Racine (1972a,b). These findings indicate that RK-induced kindled state is persistent.

Since it is well documented that the amygdala represents an epileptic focus that spreads its activity toward other brain regions such as motor cortex and hippocampus (Shi et al., 2007), we assessed the EEG activity from ipsi and contralateral M1 cortex during RK protocol. As shown in Figure 1C, epileptiform activity progressed bilaterally in the motor cortex, in parallel with the EEG activity recorded from BLA. In both ipsi and contralateral M1 cortex, the emergence of the first AD occurred with a time lag related to the first AD in BLA, which may be attributed to the propagation of ictal discharges from BLA to corticolimbic regions (Shi et al., 2007). This delay was slightly greater in the contralateral than the ipsilateral cortex; nevertheless the ending of the first ADs in both BLA and cortex occurs simultaneously. Starting from R3 a variable number of secondary ADs emerged after the first AD, showing lower amplitude and duration than the first one. Interestingly, a greater number of secondary ADs were observed in M1 cortex compared to BLA recordings (BLA, 0–1 ADs; M1 cortex, 1–3 ADs), which suggests that this activity was originated from a new epileptogenic focus recruited by the amygdaloid complex. Taken together, these findings demonstrate that epileptic activity induced by chronic RK protocol progresses gradually with the same EEG and behavioral characteristics as traditional SK protocol, propagating bilaterally from amygdala to motor cortex in both cerebral hemispheres.

### Temporal course of Racine stages in RK and SK protocols

**Figure 2:**
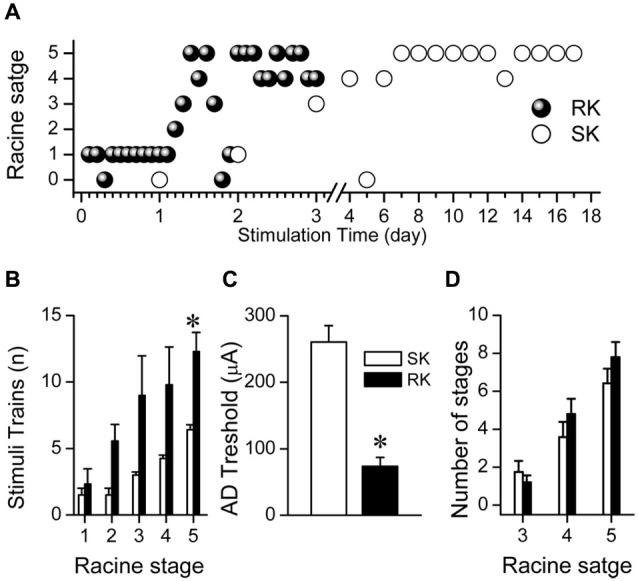
**RK rats reach kindled state faster than SK rats.** **(A)** Representative progression of Racine stages in two rats subjected to RK or SK protocols. **(B)** Number of trains required to reach every Racine stage for each protocol. **(C)** Average AD thresholds for each protocol. **(D)** Average number of total amount for Racine stages 3, 4 and 5, induced by both protocols (*t*-test, * *p* < 0.05, *n* = 9 in RK and *n* = 5 in SK).

Having established the EEG patterns induced by RK protocol, we analyzed the temporal course of motor behavior related to the stimulation properties of RK and SK protocols. Even though both groups showed a similar pattern of kindling progression, the number of days to reach each Racine stage for RK protocol was at least five times lower than for SK protocol (i.e., 1 day to reach R3 in RK vs. 5 days in SK; Figure 2A). However, a variable number of post-ictal inhibiton (PI) periods–described as the absence of ADs and/or convulsions in spite of regular stimulation- were exhibited in both RK and SK rats after a severe seizure. These episodes began to be more evident from R3. The number of trains required to reach every Racine stage was significantly greater in RK protocol than SK (Figure 2B). The maximum differences were observed at R3, which was reached with three times more trains than the SK protocol. Although the first R5 stage was reached around the 2 nd day in RK and 9–15 days in SK, the number of trains to achieve R5 in RK was about the double than in SK protocol (i.e., 12.3 ± 1.4 trains and 6.4 ± 0.4 trains, respectively; Figures 2A,B). The differences in the number of repetitive stimuli required for each protocol can be due to the lower intensity of stimulation required by RK protocol. In accordance with what was mentioned above, the average threshold to evoke ADs was significantly lower in RK than in SK (73.50 ± 13.71 μA and 260 ± 24.49 μA; *n* = 12 and *n* = 7; *p* < 0.001, respectively; Figure 2C). These results suggest that the epileptogenesis induced by RK protocol requires a temporal course about six times less than SK to reach the kindled state, progressing gradually and in similar fashion than SK protocols, reaching the same number of generalized seizures (i.e., R4 and R5) in both protocols (Figure 2D).

### ADs duration and seizure severity in RK and SK protocols

**Figure 3:**
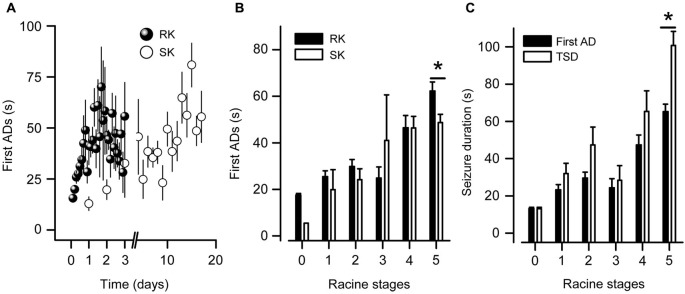
**ADs durations in kindled state from RK rats are longer than SK rats.** **(A)** Temporal progression of the first AD duration recorded in BLA of RK and SK rats. **(B)** First AD duration mean values as a function of Racine stage. Notice that in only one case a RK rat displayed ADs without behavioral manifestation (i.e., Racine stage 0). **(C)** Seizure durations corresponding to first AD and first plus secondary ADs (TSD) recorded in M1 cortex induced by RK protocol. Notice that at R5, a variable number of secondary ADs following the first one were observed, in which tonic-clonic convulsions were absent (*t*-test, * *p* < 0.05, *n* = 9 in RK and *n* = 5 in SK).

Since the Racine scale has been used as the main parameter to evaluate kindling development (Racine, 1972b; Lothman et al., 1985; Behr et al., 2000), we assessed the ADs’ parameters (duration and number) as a function of Racine stages. As shown in Figures 3A,B, AD duration progressively increased until reaching maximum values at the second day of the RK protocol, corresponding to R4-R5 stages (46.5 ± 5.2 s and 62.3 ± 3.8 s, respectively). SK group reached similar AD duration at the same stages (46.4 ± 4.9 s in R4 and 48.7 ± 3.6 s in R5) but around the ninth day of stimulation. In M1 cortex, a variable number of secondary ADs frequently emerged after the first one, showing lower amplitude, frequency and duration. TSD (see Section Methods) in kindled state exceeded the duration of the first AD in about 53% (Figure 3C). These results show that the AD duration increases in parallel with Racine stages during RK protocol, reaching values similar to those exhibited in SK group.

### Reactive astrogliosis and neuronal excitability in slices from fully kindled rats

**Figure 4:**
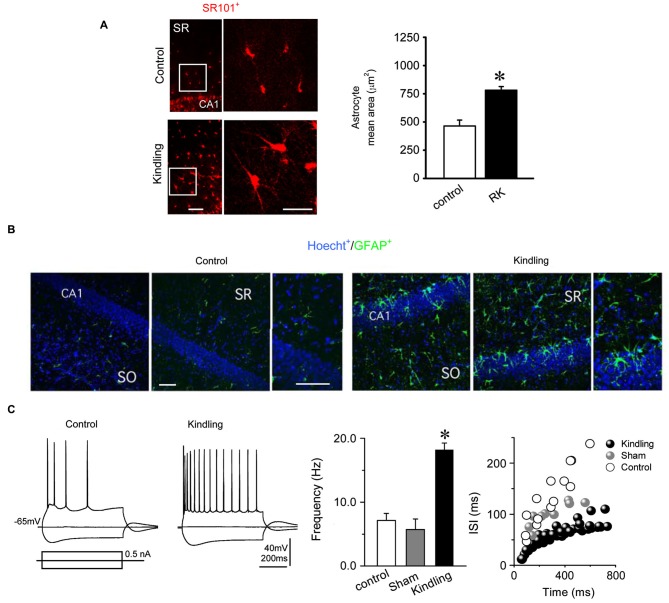
**Reactive hypertrophic astrocytes and electrophysiological neuronal alterations induced by the RK protocol.** **(A)** (Left) Astrocytes identified as SR101 positives cells in stratum radiatum (SR) of the hippocampus showed a higher mean area in kindled animals (right, bars) indicating hypertrophic morphology. **(B)** Immunohistochemical staining of the hippocampus for GFAP (green), a specific marker of astroglial processes; and Hoecht (blue), a nuclear marker. Kindled rats showed an increased GFAP immunoreactivity throughout the hippocampus compared to control rats (388% in SR, and 205% in SO). Noticeably, similar levels of Hoechst in both experimental conditions were found (78% in SR and 88% in SO; *n* = 8 slices in RK and control). **(C)** (Left) Representative voltage membrane responses from control and kindled CA1 pyramidal neurons, evoked by the injection of hyperpolarizing and depolarizing current pulses (±0.5 nA; 750 ms), recorded at −65 mV imposed membrane potential. (Right) Mean frequencies and inter-spike interval (ISI) of AP discharges evoked by pulses of +0.5 nA (Mann Whitney test, * *p* < 0.05; *n* = 5 in RK and *n* = 3 in control; calibration bars: 100 μm, for all images).

One of the greatest limitations of rapid kindling protocols described until now is that the brain structure used to induce epileptogenesis is also employed to perform electrophysiological and/or inmunohistochemical studies. This fact makes it difficult to differentiate cellular and molecular changes related to surgical trauma and direct electrical stimulation from those caused by epilepsy itself. In order to avoid these problems and to confirm the spreading of epilepsy, we set out to evaluate whether some of the cellular changes more widely accepted as a hallmark of epileptic tissue, such as astrogliosis and neuronal hyperexcitability (Binder and Steinhäuser, 2006; Schulz et al., 2012), were observed in brain structures that receive strong inputs from the amygdala. To accomplish the foregoing, epilepsy-related morphological and functional changes were assessed in hippocampal slices obtained from rats in kindled state induced by RK protocol. SR101-labeled cells with morphological appearance and location of astroglial cells were identified in all hippocampal regions including CA1, CA3 fields and hilar region of dentate gyrus as well as in all cortical layers (Figure 4A). In hippocampal slices from kindled rats, astrocytes showed a pronounced elongation of their processes, which were thicker and more sinuous than the observed in the control group (Figure 4B). In stratum radiatum as well as in cortical layers 2/3 and 5, mean area of SR101 positive cells was larger in the kindled than in the control group, suggesting astroglial hypertrophy (Kindled, 781.5 ± 32.5 10 μm^2^, *n* = 31 vs. Control, 464.1 ± 52.7 μm^2^, *n* = 31; Figure 4B). Hypertrophic astrocytes were always found bilaterally in all five kindled rats analyzed, but never in control or sham rats. Next, we evaluated immunoreactivity to GFAP, widely reported as a hallmark of astrogliosis (Wetherington et al., 2008). In CA1 field of kindled rats hippocampal slices, hypertrophic astrocytes located in both stratum oriens (SO) and radiatum (SR) showed mean values of GFAP relative area and staining intensity higher than control (Figure 4B). By using the nuclear marker Hoechst, we found no differences either in relative area or staining intensity between kindling and control slices (Figure 4B), which indicates that the number of neurons and/or glial cells in fully kindled rats did not change significantly. Taken together, these results indicate that the RK protocol induces strong reactive astrogliosis in all hippocampal formation, characterized by cellular hypertrophy and no neuronal loss or astroglial hyperplasia.

In hippocampal slices from RK group, CA1 pyramidal neurons exhibited mean resting membrane potential values of –71.5 ± 6.3 mV without significant differences in relation to sham and control groups (Input resistance, Control: 110.4 ± 15.1 MΩ vs. Kindling: 123.6 ± 24.0 MΩ). Figure 4C shows representative responses of CA1 pyramidal neurons from control and kindled group evoked by depolarizing and hyperpolarizing current pulses. When depolarized, they fired repetitive action potentials (APs; 76.3 ± 7.1 mV in amplitude and 1.2 ± 0.6 ms in duration), which were essentially identical to those recorded from sham and control groups. In pyramidal cells of RK group, the AP threshold was −49.3 ± 1.3 mV, similar to control and sham groups (−50.1 ± 0.5 mV and −45.2 ± 0.6 mV, respectively).

By using the same depolarizing current pulses and imposed membrane potential (+0.5 nA; 750 ms; −65.0 mV membrane potential), we compared the neuronal firing properties between groups. Neurons of the RK group showed a lower rate of APs accommodation than the control group (Figure 4C). Consequently, the average firing frequency was significantly higher in the RK group than in the control and sham groups (18.2 ± 1.1 Hz vs. 7.1 ± 1.1 Hz and 5.7 ± 1.6 Hz; *n* = 12, *n* = 4 and *n* = 6, respectively; Mann Whitney test, *p* < 0.05; Figure 4C). Similarly, interspike interval (ISI) of repetitive APs firing was lower in the RK group than in the control and sham groups (Figure 4C). These results show that the RK protocol induced an increase in the excitability of CA1 pyramidal neurons characterized by a raised firing rate.

Thus, these findings confirm the effectiveness of our RK protocol by corroborating that the amygdala stimulation is sufficient to generate epilepsy-related morphological and functional changes in all hippocampal formation. These changes can be directly attributed to the pathophysiological plastic process behind epilepsy development and establishment, because there was no surgery or direct electric stimulation over the hippocampus.

## Discussion

Our RK protocol provides a reproducible method for fast induction of experimental epileptogenesis in adult rats while keeping the subthreshold stimulation and gradual progression of epileptic activity of SK protocols.

Unlike previous abbreviated protocols in which the initial stimulus had suprathreshold intensity (Lothman et al., 1985; Fournier et al., 2010), in this new RK protocol subthreshold and subconvulsive stimulation intensity is sufficient to reach the kindled state in 3 days (20% of the time needed by SK protocols), persisting at least for 1 month (Elmér et al., 1998; Musto et al., 2009). In our protocol, direct stimulation of the amygdaloid complex acts as the epileptogenic focus from where epileptic activity spreads toward projection areas that receive amygdala inputs, including hippocampal formation, which exhibited epilepsy-related cellular changes (Lothman et al., 1985; Ikegaya et al., 1996; Aronica et al., 2000; Valentine et al., 2004; Musto et al., 2009). Moreover, the progressive increase in the ADs duration showed that the use of stimulation intensities below the ADs threshold effectively promotes the long-term plasticity described in the kindling model (Racine, 1972a; Abe, 2001; Schubert et al., 2005) minimizing the cellular damage attributable to excessive electrical charge utilized in other protocols (Lothman et al., 1985, 1989; Von Bohlen und Halbach et al., 2004; Fournier et al., 2010).

### RK Protocol parameters

In this study we employed sub-threshold stimulation intensities during the protocol, reducing by 20% the minimal current intensity to evoke an AD, whereas the stimulus train duration was extended to 10 s compared to SK protocol. This choice is based on two premises: (1) supra-threshold intensities can damage the tissue surrounding the electrode and do not abbreviate kindling progression and consolidation (Goddard et al., 1969; Racine, 1972a; Lothman et al., 1989); and (2) sub-threshold intensities represent a more physiological stimulation, reducing the damage on the stimulated structure as well as the ADs threshold, which reproduces the gradual enhancement of neuronal excitability observed in SK protocols (Racine, 1972a,b).

Traditionally, kindling protocols have employed one stimulation train per session (i.e., one stimulus per day), probably to ensure gradual kindling progression (Goddard et al., 1969; Racine et al., 1973). In agreement with previous reports (Goddard et al., 1969; Lothman et al., 1985), we observed that the RK progression is not possible with an inter-stimuli interval shorter than 20 min (Data not shown); hence, we used a 20-min inter-stimuli interval. Often, in both SK and RK protocols, some convulsive behavior with lower Racine score (i.e., R1–R2) and shorter or absent ADs were observed between highest Racine scores (i.e., R3–R5; Figure 2A). This apparent discontinuity known as post-ictal inhibition (PI) has been described as a period of inhibition generated after the occurrence of generalized seizures. The degree of its inhibition is proportional to the previous motor recruitment; however, the presence of ADs itself can generate mild inhibition (Mucha and Pinel, 1977; Löscher and Köhling, 2010). PI has been attributed to several mechanisms increasing inhibitory activity, such as a transient rise in the levels of adenosine (Francis and Fochtmann, 1996). PI can be overcome by using supra-threshold stimulation intensities or by increasing the inter-stimuli interval (Racine, 1972a; Peterson et al., 1981; Lothman et al., 1985; McIntyre et al., 1987). Despite this apparent lack of progression in motor seizure severity, once these PI periods finish, the AD duration progressively increases with stimulation. Therefore, these episodes were not considered as a regression in kindling development.

Although the rats reached the kindled state, spontaneous convulsions were not observed, even in those rats that presented several ADs per stimulus, suggesting that this RK protocol represents a valid model for epileptogenesis rather than ictogenesis studies. However, in our conditions spontaneous seizures should be achieved by increasing the number of stimulation sessions (Michael et al., 1998; McIntyre et al., 2002; Morimoto et al., 2004).

### Seizure propagation through the brain

Often, experimental studies in chronic epilepsy models have been carried out in the same brain structure that was stimulated to generate the epileptic focus (Aronica et al., 2000; Valentine et al., 2004). In our RK protocol, ADs initially observed in stimulated BLA -the kindling site- were later recorded in cortical regions due to bilateral spreading of the epileptiform activity as showed by the time lag between the ADs recorded in BLA with respect to M1 cortex. Similar spatiotemporal sequence of ictal discharges propagation has been described in amygdala kindling induced by SK protocol, suggesting that ADs from amygdala arrives first at cortex and then spread to the hippocampal formation (Shi et al., 2007). As shown by Shi et al. (2007), during seizures there are massive hippocampal synchronized spikes in correlation with cortical activity, which is consistent with our results. Tight bilateral hypersynchronization described in hippocampal formation during kindling seizures strongly suggests that in our protocol, the bilateral ADs recorded in M1 cortex are also a reflect of the epileptiform activity during seizures in the hippocampus (McIntyre et al., 1987; Allen et al., 1992; Ikegaya et al., 1996).

These findings open an interesting possibility of carrying out epileptogenesis studies in those projection areas from initial epileptic focus, such as cortical and mesolimbic regions (i.e., basal ganglia), in which none of the characteristic cellular changes of epileptogenesis can be attributable to mechanical and electrical damage *per se*, as occurs in a stimulation site (Goddard et al., 1969).

### Functional and morphological consequences of the RK protocol

In this work we showed that amygdala RK protocol induces the main morphological and functional changes in hippocampal formation described both in SK protocols and in resected tissue from human patients suffering temporal lobe epilepsy (TLE), including neuronal hyperexcitability and astrogliosis (Bertmam and Lothman, 1993; Behr et al., 2000; Albensi et al., 2007). It has been reported that neuronal excitability is increased in acute and chronic pharmacological models of epilepsy, which has been attributed to downregulation of the after-hyperpolarization K^+^ currents (Fernández de Sevilla et al., 2006; Schulz et al., 2012). These K^+^ currents are implicated in the accommodation and control of APs frequency. Our intracellular recordings in CA1 pyramidal neurons from kindled hippocampal slices confirmed an increase in neuronal excitability, characterized by an increase of discharge frequency and a decrease of accommodation, which is consistent with previous reports (Behr et al., 2000; Schubert et al., 2005; Schulz et al., 2012). On the other hand, hypertrophic GFAP^+^ astrocytes were found bilaterally in the hippocampus, showing strong reactive astrogliosis, which can be directly attributed to the epileptogenesis process, because there was not surgical intervention or electric stimulation over these areas. Unlike others (Cavazos and Sutula, 1990; Hosokawa et al., 1995), under our conditions the astroglial hypertrophy was observed in absence of both hyperplasia and neuronal loss. In agreement with previous (Khurgel et al., 1995), our results suggest that neuronal loss is not a prerequisite for the establishment of an epileptic state, and reactive astrogliosis could be induced by factors associated with abnormal neuronal activity more than neuronal degeneration.

Taken together, these findings demonstrate that epileptic activity induced by the RK protocol progresses gradually with the same EEG and behavioral characteristics as the traditional SK protocol, propagating bilaterally from amygdala to several projection areas, including hippocampal formation. Moreover, hippocampal neuronal hyperexcitability and astrogliosis demonstrated that the epileptogenesis process affects amygdala projection areas; thus allowing the study of epilepsy using structures different from those stimulated and surgically intervened.

In conclusion, this RK protocol represents a new variant of the chronic epileptogenesis model in freely moving rats, short in duration and highly reproducible, which permits the study of the morphological and electrophysiological alterations in main projection areas from BLA, including hipocampal formation and motor cortex.

## Conflict of interest statement

The authors declare that the research was conducted in the absence of any commercial or financial relationships that could be construed as a potential conflict of interest.
